# Palliative care in rural, remote, and northern communities: a scoping review

**DOI:** 10.1186/s12904-026-02071-7

**Published:** 2026-03-19

**Authors:** Natasha Magyar, Katherine Kortes-Miller, Lynn Martin

**Affiliations:** 1 School of Social Work, Lakehead University, 955 Oliver Road, Thunder Bay, ON P7B 5E1 Canada; 2 Centre for Education and Research on Aging and Health, Lakehead University, 955 Oliver Road, Thunder Bay, ON P7B 5E1 Canada; 3 Department of Health Sciences, Lakehead University, 955 Oliver Road, Thunder Bay, ON P7B 5E1 Canada

**Keywords:** Palliative, End-of-Life, Rural, Remote, Northern, Life-limiting disease, Barriers, Facilitators

## Abstract

**Objective:**

To review and analyze existing literature on palliative care in rural, remote, and Northern communities and consider the gaps that remain in research in order to guide the direction of the current project.

**Introduction:**

Like other areas of healthcare, persons living in rural, remote, and Northern regions face unique barriers when accessing palliative care in their communities.

This scoping review provides an analysis of the current literature addressing palliative care in rural, remote and Northern regions globally and identifies themes in existing literature to determine areas of need for future research.

**Method:**

Primary studies published in English in peer-reviewed journals between January 1, 2010 and February 2024 were identified CINAHL, ProQuest, PubMed, and OMNI.

**Results:**

A total of 53 studies were eligible for data extraction. The existing literature identifies several common barriers and facilitators to accessing palliative care. Identified barriers include travel and cost of accessing care, policy issues, lack of communication, lack of knowledge/education. On the other hand, the identified facilitators include collaboration, advanced care planning, specialized education and training of care partners, utilization of telemedicine, and the use of volunteers.

**Conclusion:**

Suggestions for addressing barriers and leveraging facilitators to palliative care in rural, remote, and northern locations throughout Canada were presented. Future research should build upon this knowledge to guide positive changes towards the shared goal of equitable access to palliative care for all Canadians, regardless of where they live.

**Supplementary Information:**

The online version contains supplementary material available at 10.1186/s12904-026-02071-7.

## Introduction

A palliative approach to care is one that meets the needs of people with serious or life-limiting illness and their family in a practical and holistic way (e.g., symptom and pain management; social, emotional, spiritual, psychological support; practical support) and begins as early as possible in the disease process [1]. The demand for palliative care is expected to increase worldwide as the age of our population and the prevalence of chronic disease continue to rise. In 2018, the Canadian government introduced the Framework on Palliative Care [1] that outlines a standard of care and provides a guideline for palliative care stakeholders to work toward. In Health Canada’s five-year progress report [2], key areas where continued efforts are needed were noted, including increased capacity and improved access to palliative care in rural, remote, and Northern regions.

Persons living in rural, remote, and Northern regions face unique barriers when accessing palliative care [3, 4]. Rural populations tend to be older and more likely to live with chronic illnesses, yet remain less likely to have access to timely, specialized palliative care [5, 6]. Due to limitations regarding staff shortages, education and training, and resources, implementation of comprehensive palliative care programs in rural, remote, and Northern areas is challenging [7, 8]. To improve access and help facilitate building community capacity, further research is needed in relation to palliative care needs in these communities.

This scoping review aims to identify current knowledge of palliative care in rural, remote, and Northern communities to understand current issues facing persons, families, providers, and palliative care and health systems. It will identify themes in existing literature and identify gaps in knowledge that warrant further research.

## Methods

This scoping review followed Arksey and O’Malley’s [9] five stage approach: (1) identify the research question, (2) identify relevant studies, (3) select studies for data extraction, (4) extract and chart data, (5) summarize and report results and the Preferred Reporting Items for Systematic reviews and Meta-analyses extension for Scoping Reviews (PRISMA-ScR) critera. The study protocol has not been published.

### Eligibility criteria

Our search strategy was developed in consultation with a librarian. Peer-reviewed primary studies published in English between January 2010 and February 2024 were eligible for inclusion. Reviews (narrative, scoping, systematic) and expert opinion pieces were excluded.

### Information sources

We performed advanced searches in CINAHL, ProQuest, PubMed, and OMNI using relevant terms and medical subject headings (MeSH).

### Search

Keywords included palliative, end-of-life, end-stage, aid in dying, hospice, terminal care, and life-limiting illness were used with the Boolean phrase “OR” to ensure that as many terms used to describe palliative care were utilized. These terms were paired using “AND” with rural, remote, or Northern. For example, the CINAHL search focused on full text articles with keywords palliative OR end of life OR aid in dying OR hospice OR terminal OR end stage AND rural OR remote OR northern in the title; we also excluded reviews (scoping reviews OR reports OR literature reviews) and articles focused on children (i.e., exclude pediatric).

### Selection of Sources of Evidence

#### Participant

We considered studies that reflected a variety of perspectives, for example, people with lived experience (PWLE) (e.g., receiving palliative care in rural, remote, or northern regions), informal carers, healthcare professionals, as well as those based on secondary analysis of health records.

#### Context

The focus of the studies should be on palliative care in rural, remote, or northern regions, but there were no restrictions based on setting (e.g., inpatient hospital, palliative care program, hospice).

#### Data charting

We developed a data extraction tool using Excel. Following the initial review of titles and abstracts by the primary and last authors, the data extraction of included articles was conducted by the first author and two research assistants; the last author reviewed five extractions from each to ensure quality and consistency of extractions.

#### Data items

The following information was extracted from eligible articles: author, year, country, study aim, design, sample size, participant characteristics, terminology and definitions used related to palliative care and rurality, and results. We also noted authors’ study limitations and suggestions for future research, though this was not included in the extraction table.

#### Synthesis of results

The authors relied on thematic analysis to identify themes throughout the existing literature. Once codes (labels) were determined, they were used to identify patterns, categories and themes across the literature. The codes were established by the first author and reviewed by both co-authors.

## Findings

### Selection of sources of evidence

**Fig. 1:**
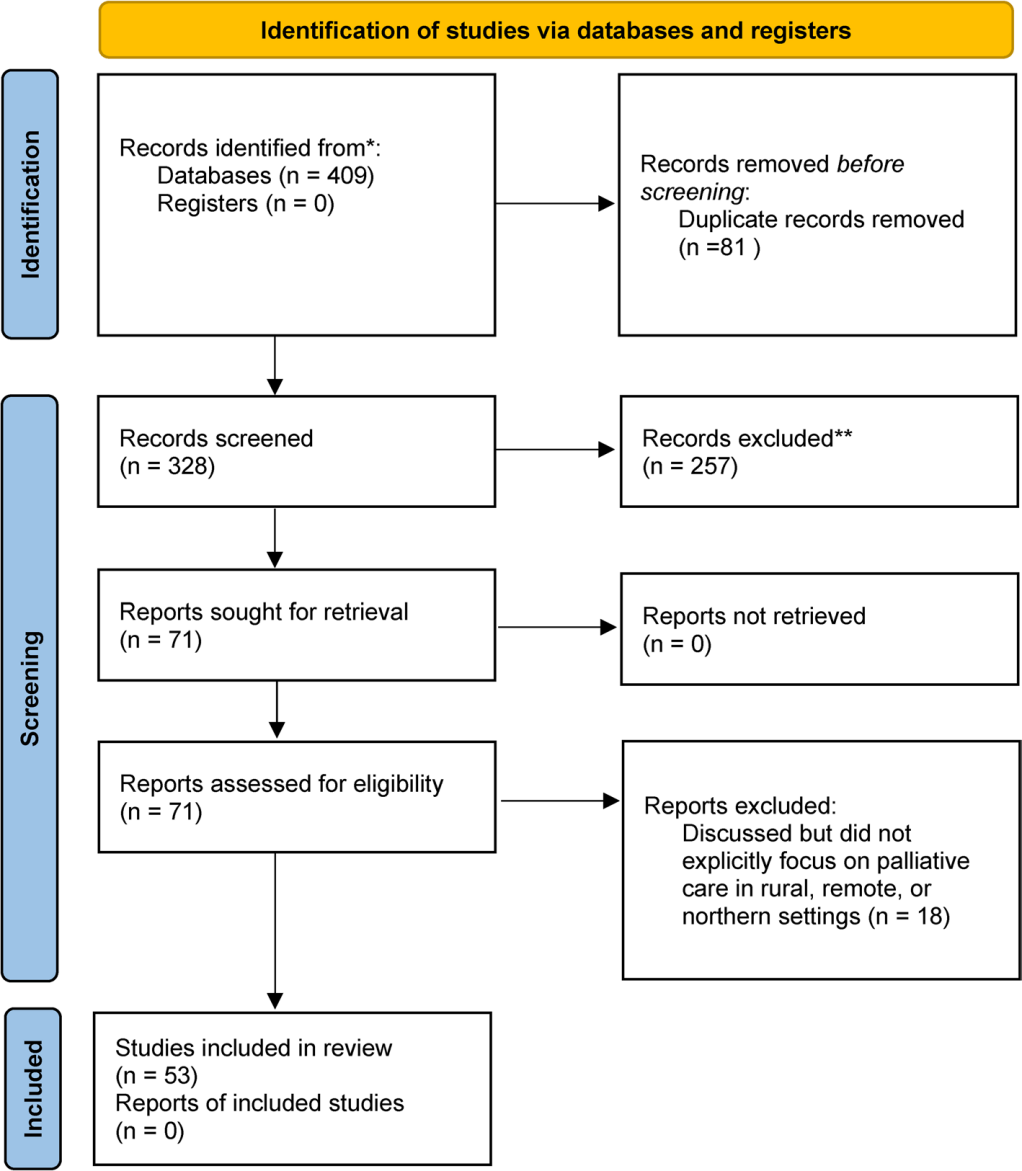
Flow chart of scoping review results

After removal of duplicates, a total of 320 articles were identified. Following review of titles and abstracts, 63 articles remained eligible for full review and data extraction. After full review, ten studies were eliminated for lack of explicit focus on palliative care services, leaving 53 studies (Fig. 1).

#### Characteristics of sources of evidence

This section describes study characteristics based on the data extraction (Table 1).

**Table 1:** Key ites from data exntrmactio

References	Country	Study design	Participants	Sample size	Participant characteristics	Term used	Findings - Themes	Findings - Subthemes	
[10]	Miller et al. (2021)	Australia	Qualitative	Informal carers	9	Sex	PC, EOL	Barriers	Travel/Expense
[11]	Wilkinson et al. (2015)	Australia	Qualitative	Records: Informal carers	223 pre; 192 post	Age, Sex	PC, terminal care	Facilitators	Education & Training
[12]	Carey et al. (2017)	Australia	Quantitative	Records: financial records, admission records	17	Age, Sex, Aboriginal and Torres Strait Islander status	PC	Facilitators	Community Based Care
[13]	Chukwusa et al. (2019)	United Kingdom	Quantitative	Records: deceased patient records (drive time to care)	430,467 (deaths)	Age	PC, EOL	Barriers	Travel/Expense
[14]	Pesut et al. (2010)	Canada	Mixed methods	PWLE	15	Age, Sex	PC	Barriers	Travel/Expense
[15]	Wilson et al. (2012)	Canada	Mixed methods	PWLE	Phase 1: 17,798; Phase 2: 108; Phase 3: 11	Age, Sex, Employment status, Relationship	EOL	Barriers	Travel/Expense
[16]	Leng et al. (2019)	China	Quantitative	Informal carers	792	Geographic place of residence, socio-economic background, marital status, age, sex, treatment decisions, healthcare utilization, healthcare costs, money borrowed/owed for care	EOL, hospice	Other: Cost analysisIdentified barriers and facilitators within analysis	Age/Socioeconomic background/location and age
[17]	Dongre et al. (2012)	India	Quantitative	PWLE	900	Age, Sex, Education, Religion, Occupation, Socioeconomic status, Marital status, Family type	PC	Facilitators	Education & TrainingCommunity Based Care
[18]	Howell et al. (2011)	Canada	Mixed methods	PWLE	95	Age, Sex, Marital status, Income	PC	Facilitators	Collaboration
[19]	Berke et al. (2023)	USA	Quantitative	HCPs	158	Age, Race, Profession	PC	Facilitators	Education & training
[20]	Del Rosario et al. (2019)	USA	Quantitative	Records: Veterans’ AffairsPWLE	66,958	Age, Sex, Race	EOL	Facilitators/Barriers	Identifies access, attitudes/values, rurality as potential barriers/facilitators for use of hospice care
[21]	Jones (2021)	USA	Quantitative	Records: Admission records and satisfaction scoresPWLE	1,175	Ethnicity	Hospice	Facilitator	Spirituality/religious component to care
[22]	Ko et al. (2020)	USA	Qualitative	Informal carers	28	Age, Sex, Race, Relationship to person	Hospice	Barriers Facilitators	Communication; Knowledge ACP
[23]	Fletcher et al. (2016)	Australia	Qualitative	HCPs	55	Age, Sex, Occupation	Advanced care planning	Facilitators	ACP
[24]	Veillette et al. (2010)	Canada	Qualitative	HCPs, Healthcare administrators, Municipal leaders, Professionals	36	Age, employment status	Good death	FacilitatorsBarriers	Communication, Knowledge (ACP), CommunityTransportation, access to care
[25]	Lam et al. (2018)	Australia	Mixed methods	HCPs	109	Age, Sex	Advance care planning	Barriers Facilitators	Communication ACP
[26]	Odgers et al. (2018)	Australia	qualitative	PWLE	12	Sex	EOL	Barriers	Communication
[27]	Lomenick et al. (2021)	USA	Quantitative	Records: Administrative cost dataPWLE	6200 total patient days of care	None specified	Hospice care	Facilitators	Telemedicine
[28]	Taylor et al. (2018)	United Kingdom	qualitative	HCPs, Researchers, Civil society organizations, Business partners	39	Not specified	Hospice Care	Facilitators	Telemedicine
[29]	Aregay et al. (2023a)	Ethiopia	Qualitative	HCPs, Researchers	42	None specified	PC	Barriers	Policy issues
[30]	Herce et al. (2014)	Malawi	Mixed methods	Records & collected data PWLE & Stakeholders	47	Age, Sex	PC	Facilitators	Collaboration
[31]	Koski et al. (2017)	Canada	Qualitative	HCPs, Community members	20	None specified	Palliative, Palliation	Facilitators	Collaboration, communication, knowledge translation
[32]	Lalani & Cai (2022)	USA	Qualitative	HCPs	15	Age, Sex	Palliative, Palliation	Barriers Facilitators	Communication Education & training
[33]	Nadin et al. (2018)	Canada	Mixed methods	Records & collected dataPWLE	22	Not specified	PC	Facilitators	Community Based Care
[34]	Näppä et al. (2023)	Sweden	Qualitative	HCPs	10	Age, Sex	PC	Barriers	Communication, Training & education & Collaboration
[35]	Prajitha et al. (2023)	India	Qualitative	HCPs		Not specified	PC	Barriers	Knowledge
[36]	Winter et al. (2020)	Canada	Mixed methods	PWLE	770	Sex	PC	Barriers	Travel/Expense
[37]	Bonsignore et al. (2018)	USA	Mixed methods	PWLE	101	Age, Sex	PC	Facilitators	Telemedicine
[38]	Koczwara et al. (2010)	Australia	Program development	HCPs	769	None specified	Palliative, Palliation	Facilitators	Education & training
[39]	Pesut et al. (2011)	Canada	qualitative	HCPs, PWLE, Families	95	Sex	PC	Barriers	Travel/Expense
[40]	Pesut et al. (2017)	Canada	mixed methods	PWLE	25	Age, Sex, Residence	PC, Palliative approach	Facilitators	Collaboration
[41]	Larson et al. (2021)	USA	Qualitative	Community Leaders	15	Age, Sex	PC, EOL	Barriers Facilitator	Personal beliefsKnowledge
[43]	Raziee et al. (2017)	Canada	Quantitative	Records: Administrative DataPWLE	17,649	Sex	Palliative, Palliation, EOL	Barriers	Rurality impacts place of death regardless of income level
[43]	Reed et al. (2018)	Australia	Mixed methods	HCPs	77	Sex	PC, EOL	Facilitators	Community Based Care
[45]	Jack et al. (2011)	Uganda	Qualitative	PWLE, HCPs, Volunteers	64	None specified	Hospice, PC	Facilitators	Volunteers
[44]	Crooks et al. (2018)	Canada	Mixed methods	HCPs, Informal carers	40	None specified	PC	Facilitators	Volunteers
[46]	Johns et al. (2019)	Australia	Qualitative	PWLE, Families, HCPs	38	Sex	Terminally ill, PC	Barriers	Policy issues; Knowledge
[47]	Johansen & Ervik (2022)	Norway	Qualitative	HCPs	52	Age, Sex	PC	Barriers	Communication
[48]	Munday et al. (2018)	India	Mixed methods	Hospital Administrators		Not clear	PC	Facilitators	Collaboration
[49]	Conlon et al. (2019)	Canada	Quantitative	Records: Linked health databasesPWLE	129,107	Age, Sex, Income quintile	PC	Barriers	Community Based Care
[55]	Walter et al. (2018)	Germany	Quantitative	Records: Patient recordsPWLE	12,929 patients in initial groupSubgroup: 7707	Place of residences based on district type (rural, urban, remote, ect.), age, gender, diagnosis, treatment,	EOL, PC, Supportive care	Facilitators Barriers	GP Knowledge Access, travel
[50]	Crooks et al. (2010)	Canada	Qualitative	HCPs, Informal carers	31	None specified	PC	Barriers Facilitators	Policy issues Collaboration
[51]	Ding et al. (2019)	Australia	Qualitative	HCPs, Professionals	37	Age, Profession	PC	Barriers	Policy issues
[52]	McVeigh et al. (2019)	USA	Qualitative	HCPs	16	None specified	PC	Barriers	Communication
[53]	Aregay et al. (2023b)	Ethiopia	Qualitative	HCPs	38	None specified	PC	Barriers	Knowledge; Policy issues
[54]	McLouth et al. (2023)	USA	Quantitative	PWLE	77	Sex	PC	Barriers	Knowledge
[56]	Mitchell et al. (2016)	Australia	Mixed methods	PWLE	62	Age, Sex	PC	Facilitators	Collaboration (ACP)
[57]	Potts et al. (2019)	India	Qualitative	HCPs	10	Age, Sex	PC	Facilitators	Collaboration & Education
[58]	Watanabe et al. (2021)	Japan	Quantitative	Organizations	264	Not specified	PC	Facilitators	Collaboration
[59]	Duggleby et al. (2016)	Canada	Consensus (Delphi)	Researchers, Knowledge users, Stakeholders	30	None specified	PC, Advanced illness	Facilitators	Advocacy, collaboration, community-based care
[60]	Klinger et al (2012)	Canada	Quantitative	Records: Economic Records PWLE	95	Sex	EOL	Facilitators	Collaboration
[61]	Weng et al. (2022)	USA	Quantitative	Organizations	17	Not specified	PC	Facilitators	Collaboration Training
[62]	Ohta & Ryu (2021)	Japan	quantitative	PWLE	96	Sex		Facilitators	Telemedicine

*PWLE *Persons with Lived Experience

*HCP *Health Care Provider

*PC* Palliative care

*EOL* End of life

#### Country

The majority of studies were conducted in Canada (*n* = 15), Australia (*n* = 11), the United States (*n* = 11). Studies from China (*n* = 1), Ethiopia (*n* = 2), Germany (*n* = 1), India (*n* = 4), Japan (*n* = 2), Malawi (*n* = 1), Norway (*n* = 1), Sweden (*n* = 1), Uganda (*n* = 1), and the United Kingdom (*n* = 2) were also identified.

#### Study design

Most studies employed qualitative methods (*n* = 23), which included interviews or observation. There were nineteen quantitative studies, many of which analyzed existing or retrospective data from healthcare and government databases. Thirteen studies employed mixed methods; one was based on a Delphi consensus building approach, and the other on program development^10^.

#### Study samples and participants

Study participants varied across the studies reviewed. Many focused on a single participant type (i.e., persons with lived experience (*n* = 11), health care professionals (*n* = 12), informal carers or family (*n* = 2), administrators (*n* = 1) and community stakeholders (*n* = 2), while some included multiple participant types that either did (*n* = 5) or did not (*n* = 8) include persons with lived experience. Three studies focused on organizational experiences, while one used geography to model communities in need of services.

Overall, the majority had fewer than one hundred participants. Qualitative samples ranged between nine [10] and 223 [11], quantitative samples between seventeen [12] and 430,467 [13], and between fifteen [14] and 17,798 [15] for mixed methods studies.

The reporting of participant demographics tended to be mixed. Only Leng and colleagues [16] provided extensive information on characteristics. A few studies presented information related to marital status [17, 18], race or ethnicity [12, 19–22], education [17], employment status or occupation [17, 19, 23, 24], socio-economic status [16–18, 25], and religion [17].

#### Palliative terminology and definitions

Several terms were used to describe target services. More than half referred to palliative care/approach and palliation, either exclusively (*n* = 31) or in conjunction with another term (*n* = 9). A few studies exclusively used other terms, including hospice or hospice care (*n* = 4), end-of-life care (*n* = 4), advance care planning (*n* = 2), and good death (*n* = 1). Overall, approximately half (*n* = 29) of the studies did not define the terminology used.

The single study that employed the term “good death” provided multiple definitions, concepts, and theories - as its goal was to understand its meaning to individuals [24]. Advance care planning was defined by both studies that used it as a discussion focused on preferences, needs, and choices for the future [23, 25].

Two of the four articles that referred to end-of-life care offered definitions. Odgers and colleagues [26] described it as a conversation that should happen about goals, preferences, and services at the end of life, whereas Del Rosario and colleagues [20] described the elements of high-quality end-of-life care, including pain management, recognition of the emotional and spiritual needs of the individual and family, and referral to specialist palliative or hospice care.

Each of the four studies that exclusively used the term hospice or hospice care defined it. All but one study explicitly noted it is an interdisciplinary approach [21, 22, 27, 28]. Jones [21] further explained that it aims to help “patients cope with sadness and anxiety” (p185). Lomenick and colleagues [27] broadly referred to hospice care as provision of “quality end-of-life care for individuals facing life-limiting illness and/or injury” (p1461), while Ko and colleagues23 described it as a wide variety of services that included “pain and symptom management, bereavement services, psychosocial and spiritual care, for families and patients”. Taylor and colleagues [28] defined hospice care as providing “physical, social, emotional, and spiritual support for people with life-shortening illness”.

Of the articles that relied solely on the terms palliative care/approach or palliation eight included that palliative care is an approach to care that addresses the physical, emotional, psychological, and spiritual well-being of a patient in their definition [29–36]. In addition, these same articles included that good palliative care aims to improve the quality of life for the patient and/or their family through meeting the patient’s needs.

The remaining articles that relied on the term palliative care/approach or palliative differed slightly but shared some commonalities. Bonsignore and colleagues [37], Koczwara et al. [38], and Pesut et al. [39] define palliative care/approach as a type of care that manages symptoms of a chronic or life-limiting illness. Koczwara et al. [38] also included that this approach aims to assist patients and their caregivers in informed decision making alongside respecting the patient’s wishes regarding their care. Pesut and colleagues [40] also emphasize that a palliative care/approach honours the patient’s wishes as to the extent possible.

Five articles used the terms palliative care and end of life care interchangeably [10, 13, 41–43], whereas one article relied on the term hospice in addition to palliative care [44]. Jack et al. [45] defines palliative/end of life/hospice care as an approach that occurs within the home. These authors attest in their definition that this type of care aims to improve patient quality of life by addressing their needs alongside relieving their symptoms.

In their definition, Miller et al. [10] focused on unpaid carers and explicitly referred to provision of financial care as being important; this was not included in any other definition. Finally, Reed et al. [43] defined it as a specific time or state of one’s disease/illness progression and suggest that palliative/end of life care is the point at which the disease is considered to be terminal.

#### Rural, remote, and northern definitions and measures

More than half of the articles relied primarily on the term rural (*n* = 37), with the remainder using the terms remote and northern either in conjunction with the term rural or as another term used interchangeably with rural. Eighteen articles did not define the term or measure used to determine rurality, or remoteness of the location of study.

The term rural was used in thirty-seven articles, and in conjunction with other terms in most remaining articles (*n* = 15). Thirty-three articles provided either a definition or measure for determining rurality. The term rural was primarily defined by population of a given area and measured by person per square kilometer or mile (*n* = 21). The remaining articles defined rurality by the distance from the nearest metropolitan area (*n* = 6), the distance to the nearest specialized care facility (*n* = 6), or a combination of these measures (*n* = 4). One article relied on the number of physicians in a geographical radius as the primary measure of rurality [32]. The term sparsely populated was also used interchangeably with rural. The threshold required to be defined as rural varied by location of study.

The term remote was used in four articles, never exclusively. It was used most often to identify a location of study as “hard to access” or as more isolated when compared to other communities designated as rural. In three articles that relied on this term, it was defined by an already established geographical designation and mainly measured by severity of isolation [12, 44, 46], in the remaining article it was defined by population density and commuting time to an urban area [39]. All articles that used the term remote, relied on a measure set by a governing body in the location of study. The terms isolated, and hard to reach were used interchangeably or instead of remote, however the definition and measure of all three terms were relatively the same.

The term northern was used in five articles to identify the geographic location and was always used in conjunction with either rural or remote [12, 41, 47–49]. While all articles that used the term northern appeared to rely on an establish national standard, only two articles provided a measurement or definition for the term, both of which explicitly stated that they were measured by a national standard or guideline in the jurisdiction of the study [48, 49].

## Synthesis of results

The objective of this review was to identify the focus and findings of existing literature on palliative care in rural, remote, and Northern communities. Two major themes were identified across study topics: (1) barriers for accessing palliative care and (2) facilitators for improving access. The following section provides a brief overview of the analysis and sub-themes within both overarching themes.

### Barriers for accessing palliative care in rural, remote and northern areas

Nineteen studies addressed barriers in accessing palliative care in rural, remote and northern areas. All recognized that lack of access to palliative services and providers was the primary cause of reduced utilization of palliative care for this population. The following barriers were also noted: travel and expense associated with accessing care outside the community [11,14,15,16,25,37,39.50], policy issues [29, 46, 50, 51], communication [22, 25, 26, 32, 34, 47, 52], and knowledge [22, 32, 35, 46, 53, 54].

#### Travel and expense of accessing care outside the community

Eight articles identified travel to access care outside the community and the associated costs as a barrier for accessing equitable palliative care. Pesut and colleagues [14] found that some people were forced to cut expenses such as groceries to afford the high cost of travel.

A Canadian study and German study highlighted that in many rural communities, palliative services simply do not exist [15, 55]. When care is available, they found that it is scattered across many places (where care transitions varied from 8 to 39 per individual), which significantly increased travel.

Chukwusa and colleagues [13] studied the drive time and place of death and found that, for those living in rural areas (compared urban areas) the distance between home and care center played a role in whether one dies at home, hospital, or in another type of care facility.

Miller and colleagues [10] found that informal carers living in rural areas face stress due to traveling to access necessary care for their loved ones, in addition to feeling burnt out and isolated.

#### Policy issues

Four articles identified issues with policy as a primary barrier. According to two articles by Aregay and colleagues [29, 53], governments still hold low priority and focus on palliative care. Many governments have adopted palliative care frameworks but draw attention to their focus on the management of curable diseases rather than holistic care models, the absence of funding and other resources required to actualize necessary care, and lack of awareness of frameworks among leaders and professionals. Ding and colleagued [51] focused on fiscal politics of palliative care frameworks. More specifically, how inappropriate payment models may contribute to discouragement of primary care providers from remaining involved in certain aspects of EOL care. Despite the existence of closer doctor-patient relationships and better care integration and collaboration in rural settings compared to urban settings, limited awareness of local resources among primary care providers constrained their effective utilization. According to Crooks and colleagues [50], politics act as barriers by creating tensions and limiting choices through the regulation of “best practices” and hierarchical structure of organizations.

#### Communication

Effective communication between patients/families and care providers and all levels of care is at the core of quality healthcare. Six articles cited communication problems as a primary barrier for accessing adequate palliative care. Lack of communication played a significant role in the breakdown of advance care planning [25, 26, 32] and has a lasting emotional and psychological effect on family members who reported feeling unprepared for the ACP process [26]. Further, lack of communication between hospitals and primary care providers acts as a barrier to palliative care, leading to delayed referrals and discharges [32, 34, 47, 52] as well as anxiety among patients and their families or caregivers [52]. In their work, Ko and colleagues [22] focused on the role of patients and family members, finding that they also played a part in communication breakdown (e.g., lack of knowledge transfer).

#### Knowledge

Because specialized services are often rendered outside the community, many care providers, family members, and patients are unaware to both the services available, and the logistics of what care can or should look like in practice. Six articles referred to lack of knowledge as a primary barrier to care.

Several studies identified lack of awareness of what palliative care is as a barrier to accessing this specialized care in rural, remote, and northern areas [22, 32, 35, 46, 54]. In fact, a large proportion of people can not differentiate between hospice and palliative care [53] or hold biases and skepticism towards the benefits of a palliative approach to care [35]. In their work, Aregay and colleagues [29] drew attention to how limited national leadership, societal norms and attitudes about chronic illness, and a lack of training and investments in palliative care have contributed to the overall lack of knowledge.

### Facilitators for improving access to palliative care in rural, remote, and northern areas

Thirty-five articles noted facilitators for improving access to palliative care in rural, remote, and Northern locations, including: Collaboration [18, 30, 40, 48, 50, 56–61], Advanced Care Planning [22–25], Volunteers [44, 45], Education & Training [19, 32, 38, 53], Telemedicine [27, 28, 37, 62], Communication [24, 31, 52], and Community Based Care [12, 17, 20, 33, 43, 61].

### Collaboration

A significant theme noted was the need for collaboration between patients and primary care physicians, and between primary care physicians and specialty care providers outside the patient’s home community. One article noted the many benefits of nurse practitioners working in a collaborative practice with primary care providers to negotiate a care plan via case conference [56], including timely initiation of care, increased follow-up, and a well-designed care plan that outlined the responsibilities of each care professional. This type of multidisciplinary collaboration could be effective in reducing patient utilization of other health care settings such as emergency rooms and hospitalizations in acute care settings [56, 61].

Pesut et al. [40] reported people and families were highly satisfied with use of a nurse navigator to facilitate services (e.g., symptom management, advance care planning, psychosocial support, education) for older adults with advanced illness living at home. Others have demonstrated the effectiveness of collaborative approach between public and private sectors [30], specialized palliative care and community services [48, 50], between community-based health workers and community services [50, 57, 59], and within a shared care program [18]. In particular, the shared care model had the potential to reduce symptom-related suffering, leading to suggestions that collaboration among interdisciplinary teams could increase the ability for patients to die at home [60]. Watanabe et al. [58] also found that collaboration between rural clinics, local hospitals, and community services also enabled people to achieve an at-home death.

### Advanced care planning

Although barriers exist to accessing advanced care planning (ACP) in rural palliative care, both formal and informal care providers have been found to be largely supportive of the process26, where some health care professionals consider it vital [23]. Factors that facilitated ACP included knowledge of EOL wishes, timing, education of and communication with primary care providers, education of patients and family members, and utilization of standardized processes to document and communicate wishes were noted as important to facilitate ACP [22–25].

### Volunteers

Two articles mentioned the benefits of volunteers to facilitate palliative care in rural, remote, and northern communities. Specifically, help with transportation, meals, and emotional support [44] as well as with physical care, practical assistance, and liaising [45] had a positive impact on patients and families.

### Education & training

Four articles cited the education and training of care professionals as facilitators. Lalani and Cai [32] identified professionals who were already trained and educated to identify needs for palliative care helped facilitate better outcomes, just as did those who promoted education among their care teams. Similarly, Koczwara et al. [38] found that education influenced approximately 75% of participants to change to the way they provided care. Both Aregay et al. [53] and Berke et al. [19] reported that education on cultural aspects of care, in addition to experience caring for people who are actively dying and advance care planning, had an important impact on outcomes.

### Telemedicine

In the context of rural, remote, and northern areas - and especially in recent years, telemedicine is heavily relied on [63]. A recent study found that it is more positively adopted in rural settings, cited in part due to the “culture” of rural medicine and the perceived benefits of telemedicine [64] (e.g., quick response, access to clinicians, efficiency and quality of care) [37]. Successful integration of telemedicine in the context of rural palliative care, in part, requires awareness, trust and familiarity, and education and training [28]. Once implemented, telemedicine has been shown to improve symptom management [37, 62], reduce travel and emergency transportation [27], emergency room use [62], and hospice transfers [37].

### Communication

Three articles clearly stated that communication was an important facilitator for care in rural, remote and northern communities. Veillette and colleagues [24] found that individuals and their families had better experiences when accessing care if communication between patient, friends, family, and healthcare providers was clear and remained effective. McVeigh and colleagues [52] also noted that clear communication regarding prognosis, and options for care contributed to better patient and family outcomes. Finally, Koski and colleagues [31] found that opening lines of communication between formal care providers and community members allowed for the provision of culturally sensitive care.

#### Community based resources

Of the thirty-five articles that discussed facilitators to care, six explicitly highlighted having access to options for community-based care as a primary facilitator of positive outcomes, including respite care facilities [12], community-managed PC [17, 33], and community-based advocacy [43]. Each of these studies suggested that community-based resources led to increased utilization of care and more positive care outcomes.

Additionally, Weng et al. [61] implemented a community capacity building program to increase access to community-based care. Their findings point towards positive outcomes when communities successfully implement and sustain palliative care programs.

## Discussion

This scoping review identified 53 studies on palliative care in rural, remote, and northern settings. Studies examined several issues (e.g., barriers and facilitators, roles, service use) from a variety of perspectives (e.g., patients, providers, family). Overall, it was clear that persons in rural, remote, and Northern regions face barriers in accessing timely and effective palliative care services.

There is a clear need for change to policy, practice, and availability of services, as well as research that evaluates such changes. In particular, policy changes at all levels were cited as needed to improve access, quality, and outcomes of palliative care in rural, remote, and northern communities [29, 53]. To be effective, rural palliative care relies on the collaborative efforts of an interdisciplinary care team. Crooks and colleagues [50] identified three types of politics that are impacting palliative care in its present state: inter-community, inter-site, and inter professional. Among the three types of politics, each carry with it risk to both the services requested but to the patients as well. On the other hand, politics also act as facilitators for care in that they promote advocacy among those involved in care.

Several studies focused on needed changes to practice - including use of informed, interdisciplinary, collaborative teams. The need to educate health care professionals on palliative care and approaches, and the roles and value of different professionals on the care team was highlighted as both important and warranting additional research. Several studies noted that providers held positive attitudes towards learning about palliative care and implementing evidence-based approaches to increase capacity for providing care locally. This is important, as they tend to have both close relationships with their patients and a deep understanding of unmet needs in the community. That said, professionals also need more information on how to best utilize the resources and technology available in their communities. The ability to leverage available resources in an effective way through collaboration and utilization of each member’s skills is paramount to the successful implementation of rural palliative care, especially in low-resource areas. In fact, collaboration was most often noted as an area for further research, with a heavy emphasis on how to best leverage existing community resources to improve access to palliative care. The need to understand the experience of health professionals providing care in the context of limited resources is important and should be more explicitly studied. Anxiety regarding the ability to meet the needs of residents is to be expected in rural, remote, and northern areas where resources are often already limited or over capacity. To ensure equitable access to care, a better understanding of professionals’ concerns is needed.

There is a clear need for additional access to quality palliative care services in rural, remote, and northern communities. In particular, additional psychosocial support for individuals, families, and caregivers was most often mentioned. Availability of respite and home-making services, as well as social work and mental health supports would greatly impact the experiences of individuals and their families. Utilization and reliance on volunteers in rural, remote, and northern communities has been well-documented. However, further research should study the social and fiscal costs and benefits at both the individual and community levels. Many noted the role that telemedicine could play in providing access to needed services, while relieving the financial strain on patients, families, and the healthcare system as a whole. Future research that focuses on understanding the short and long-term benefits to individuals and families, as well as the contexts in which its use is most beneficial is needed. In addition, given acceptability and clinical and cost effectiveness, further evaluation of telemedicine and related innovative models of care are warranted.

In the context of improving access to and the quality of care, patients provide an important perspective to understand gaps in care. However, given what people receiving palliative services are experiencing, their families and health care professionals are often the focus of studies. Inclusion of individuals as much as possible in research is needed and could be facilitated by reaching out earlier on in their journey. Often, retrospective analysis of records of clinical status and health service utilization is employed to understand the experiences of individuals at the end of life. While this allows examination of the experiences of a large number of people, the analyses conducted are often at a high level and do not consider the role of various sociodemographic factors, nor their intersectionality. When large datasets are available for analysis, consideration of sex, gender, ethnicity, race, language, disability, and other sociodemographic factors should be the standard. Further, retrospective analyses should consider individual and caregiver outcomes over time to understand the impact of different services, or lack thereof.

In Canada, family members provide approximately 80% of all palliative care that is done at home [65]; as respite and home support is limited this number is likely higher in rural, remote, and northern areas. Because of this, family members, friends, and loved ones, especially those who provide care to patients in need of palliative care services provide invaluable contributions to research in this area. By prioritizing informal care providers in the research, the lived experiences and needs of this population, including patients can be understood when translating knowledge and considering the needs for future research and changes or additions to care.

### Strengths and limitations

Our scoping review had several strengths, including mapping the focus of current research related to palliative care in rural, remote, and northern contexts, and inclusion of a large number of primary studies. Examination of terminology and definitions used for both key concepts (i.e., palliative care and rurality) was unique and showed the variability and interchangeable use of terms across studies. The review also had limitations, primarily related to exclusion of grey literature which may have led to potential publication bias in terms of the type of studies considered. However, given that much of the literature covered in this review employed qualitative designs, risk of publication bias is somewhat tempered. While we did not limit our review in terms of study aims or country, we acknowledge that most included studies are from high-income countries and focused on delivery of palliative care services rather than on system-level factors that shape delivery, for example.

## Conclusion

As populations age, and the diagnosis of life-limiting chronic illness continues to rise, the pressure on governments and healthcare systems to develop and implement palliative care programs that adequately address the needs of patients in a timely manner is expected to peak in coming years. In Canada, and around the world, rural, remote, and Northern populations face unique challenges that need to be addressed to facilitate access to quality palliative care.

While health care professionals have important insights and perspectives into the lived experience of patients and their families, the voices of individuals and families should be prioritized to truly understand the rural palliative care experience.

## Supplementary Information

Supplementary Material 1.

Supplementary Material 2.

## Data Availability

This is a scoping review. All data generated during this study are included in this published article (i.e., Summary Table).

## Abbreviations

ACP: Advance Care Planning
EOL: End of life
HCP: Health Care Provider
PWLE: Persons with Lived Experience
PC: Palliative care
