# Supplementary material for: Palliative care in rural, remote, and northern communities: a scoping review
Source: BMC Palliat Care. 2026 Mar 19;25:117. doi: 10.1186/s12904-026-02071-7 (PMC13123217; doi:10.1186/s12904-026-02071-7)
Supplement: Supplementary file 1 — Supplementary Material 1. [file 12904_2026_2071_MOESM1_ESM.docx]

Table 1. Key items from Data Extraction

| **References** | | **Country** | **Study design** | **Participants** | **Sample size** | **Participant characteristics** | **PC terms** | **Rural terms** | **Findings - Themes** | **Findings - Subthemes** |
| --- | --- | --- | --- | --- | --- | --- | --- | --- | --- | --- |
| 10 | Koczwara et al. (2010) | Australia | Program development | HCPs | 769 | None specified | Palliative, Palliation | Rural, remote | Facilitators | Education & training |
| 11 | Miller et al. (2021) | Australia | Qualitative | Informal carers | 9 | Sex | PC, EOL | Rural | Barriers | Travel/Expense |
| 12 | Wilkinson et al. (2015) | Australia | Qualitative | Records | 223 pre; 192 post | Age, Sex | PC, terminal care | Rural | Facilitators | Education & Training |
| 13 | Carey et al. (2017) | Australia | Quantitative | Records | 17 | Age, Sex, Aboriginal and Torres Strait Islander status | PC | Northern, remote | Facilitators | Community Based Care |
| 14 | Chukwusa et al. (2019) | United Kingdom | Quantitative | Records | 430,467 (deaths) | Age | PC, EOL | Rural | Barriers | Travel/Expense |
| 15 | Pesut et al. (2010) | Canada | Mixed methods | PWLE | 15 | Age, Sex | PC | Rural | Barriers | Travel/Expense |
| 16 | Wilson et al. (2012) | Canada | Mixed methods | PWLE | Phase 1: 17,798; Phase 2: 108; Phase 3: 11 | Age, Sex, Employment status, Relationship | EOL | Rural | Barriers | Travel/Expense |
| 17 | Leng et al. (2019) | China | Quantitative | Informal carers | 792 | Geographic place of residence, socio-economic background, marital status, age, sex, treatment decisions, healthcare utilization, healthcare costs, money borrowed/owed for care | EOL, hospice | Rural | Other (Cost analysis) | -- |
| 18 | Dongre et al. (2012) | India | Quantitative | PWLE | 900 | Age, Sex, Education, Religion, Occupation, Socioeconomic status, Marital status, Family type | PC | Rural | Facilitators | Education & Training  Community Based Care |
| 19 | Howell et al. (2011) | Canada | Mixed methods | PWLE | 95 | Age, Sex, Marital status, Income | PC | Rural | Facilitators | Collaboration |
| 20 | Berke et al. (2023) | USA | Quantitative | HCPs | 158 | Age, Race, Profession | PC | Rural | Facilitators | Education & training |
| 21 | Del Rosario et al. (2019) | USA | Quantitative | Records | 66,958 | Age, Sex, Race | EOL | Rural | Facilitators Barriers | Identifies access, attitudes/values, rurality as potential barriers/facilitators for use of hospice care |
| 22 | Jones (2021) | USA | Quantitative | Records | 1,175 | Ethnicity | Hospice | Rural | Facilitator | Spirituality/  religious component to care |
| 23 | Ko et al. (2020) | USA | Qualitative | Informal carers | 28 | Age, Sex, Race, Relationship to person | Hospice | Rural | Barriers Facilitators | Communication; Knowledge ACP |
| 24 | Fletcher et al. (2016) | Australia | Qualitative | HCPs | 55 | Age, Sex, Occupation | ACP | Rural | Facilitators | ACP |
| 25 | Veillette et al. (2010) | Canada | Qualitative | HCPs, Healthcare administrators, Municipal leaders, Professionals | 36 | Age, employment status | Good death | Rural | Facilitators  Barriers | Communication, Knowledge (ACP), Community  Transportation, access to care |
| 26 | Lam et al. (2018) | Australia | Mixed methods | HCPs | 109 | Age, Sex | ACP | Rural | Barriers Facilitators | Communication ACP |
| 27 | Odgers et al. (2018) | Australia | Qualitative | PWLE | 12 | Sex | EOL | Rural | Barriers | Communication |
| 28 | Lomenick et al. (2021) | USA | Quantitative | Records | 6200 total patient days of care | None specified | Hospice care | Rural | Facilitators | Telemedicine |
| 29 | Taylor et al. (2018) | United Kingdom | Qualitative | HCPs, Researchers, Civil society organizations, Business partners | 39 | Not specified | Hospice Care | Rural, remote | Facilitators | Telemedicine |
| 30 | Aregay et al. (2023a) | Ethiopia | Qualitative | HCPs, Researchers | 42 | None specified | PC | Rural | Barriers | Policy issues |
| 31 | Herce et al. (2014) | Malawi | Mixed methods | Records,  PWLE, Stakeholders | 47 | Age, Sex | PC | Rural | Facilitators | Collaboration |
| 32 | Koski et al. (2017) | Canada | Qualitative | HCPs, Community members | 20 | None specified | Palliative, Palliation | Rural | Facilitators | Collaboration, communication, knowledge translation |
| 33 | Lalani & Cai (2022) | USA | Qualitative | HCPs | 15 | Age, Sex | Palliative, Palliation | Rural | Barriers Facilitators | Communication  Education & training |
| 34 | Nadin et al. (2018) | Canada | Mixed methods | Records, PWLE | 22 | Not specified | PC | Rural, remote | Facilitators | Community Based Care |
| 35 | Näppä et al. (2023) | Sweden | Qualitative | HCPs | 10 | Age, Sex | PC | Rural | Barriers | Communication, Training & education & Collaboration |
| 36 | Prajitha et al. (2023) | India | Qualitative | HCPs |  | Not specified | PC | Rural | Barriers | Knowledge |
| 37 | Winter et al. (2020) | Canada | Mixed methods | PWLE | 770 | Sex | PC | Rural, remote, Northern | Barriers | Travel/Expense |
| 38 | Bonsignore et al. (2018) | USA | Mixed methods | PWLE | 101 | Age, Sex | PC | Rural | Facilitators | Telemedicine |
| 39 | Pesut et al. (2011) | Canada | qualitative | HCPs, PWLE, Families | 95 | Sex | PC | Remote | Barriers | Travel/Expense |
| 40 | Pesut et al. (2017) | Canada | mixed methods | PWLE | 25 | Age, Sex, Residence | PC, Palliative approach | Rural | Facilitators | Collaboration |
| 41 | Larson et al. (2021) | USA | Qualitative | Community Leaders | 15 | Age, Sex | PC, EOL | Rural | Barriers  Facilitator | Personal beliefs  Knowledge |
| 42 | Raziee et al. (2017) | Canada | Quantitative | Records | 17,649 | Sex | Palliative, Palliation, EOL | Rural | Barriers | Rurality impacts place of death regardless of income level |
| 43 | Reed et al. (2018) | Australia | Mixed methods | HCPs | 77 | Sex | PC, EOL | Rural, remote | Facilitators | Community Based Care |
| 44 | Jack et al. (2011) | Uganda | Qualitative | PWLE, HCPs, Volunteers | 64 | None specified | Hospice, PC | Rural, remote | Facilitators | Volunteers |
| 45 | Crooks et al. (2018) | Canada | Mixed methods | HCPs, Informal carers | 40 | None specified | PC | Rural, remote | Facilitators | Volunteers |
| 46 | Johns et al. (2019) | Australia | Qualitative | PWLE, Families, HCPs | 38 | Sex | Terminally ill, PC | Rural, remote | Barriers | Policy issues; Knowledge |
| 47 | Johansen & Ervik (2022) | Norway | Qualitative | HCPs | 52 | Age, Sex | PC | Rural, Northern | Barriers | Communication |
| 48 | Munday et al. (2018) | India | Mixed methods | Hospital Administrators |  | Not clear | PC | Rural, Northern | Facilitators | Collaboration |
| 49 | Conlon et al. (2019) | Canada | Quantitative | Records | 129,107 | Age, Sex, Income quintile | PC | Rural, Northern | Barriers | Community Based Care |
| 50 | Walter et al. (2018) | Germany | Quantitative | Records | 12,929 patients in initial group  Subgroup: 7707 | Place of residences based on district type (rural, urban, remote, ect.), age, gender, diagnosis, treatment, | EOL, PC, Supportive care | Rural | Facilitators  Barriers | GP Knowledge  Access, travel |
| 51 | Crooks et al. (2010) | Canada | Qualitative | HCPs, Informal carers | 31 | None specified | PC | Rural | Barriers Facilitators | Policy issues Collaboration |
| 52 | Ding et al. (2019) | Australia | Qualitative | HCPs, Professionals | 37 | Age, Profession | PC | Rural, remote | Barriers | Policy issues |
| 53 | McVeigh et al. (2019) | USA | Qualitative | HCPs | 16 | None specified | PC | Rural | Barriers | Communication |
| 54 | Aregay et al. (2023b) | Ethiopia | Qualitative | HCPs | 38 | None specified | PC | Rural | Barriers | Knowledge; Policy issues |
| 55 | McLouth et al. (2023) | USA | Quantitative | PWLE | 77 | Sex | PC | Rural | Barriers | Knowledge |
| 56 | Mitchell et al. (2016) | Australia | Mixed methods | PWLE | 62 | Age, Sex | PC | Rural | Facilitators | Collaboration (ACP) |
| 57 | Potts et al. (2019) | India | Qualitative | HCPs | 10 | Age, Sex | PC | Rural | Facilitators | Collaboration & Education |
| 58 | Watanabe et al. (2021) | Japan | Quantitative | Organizations | 264 | Not specified | PC | Rural | Facilitators | Collaboration |
| 59 | Duggleby et al. (2016) | Canada | Consensus (Delphi) | Researchers, Knowledge users, Stakeholders | 30 | None specified | PC, Advanced illness | Rural | Facilitators | Advocacy, collaboration, community-based care |
| 60 | Klinger et al (2012) | Canada | Quantitative | Records | 95 | Sex | EOL | Rural | Facilitators | Collaboration |
| 61 | Weng et al. (2022) | USA | Quantitative | Organizations | 17 | Not specified | PC | Rural | Facilitators | Collaboration  Training |
| 62 | Ohta & Ryu (2021) | Japan | quantitative | PWLE | 96 | Sex | PC | Rural | Facilitators | Telemedicine |

ACP=Advance Care Planning

EOL=End of life

HCP=Health Care Provider

PWLE=Persons with Lived Experience

PC=Palliative care
